# Health and Physical Education Preservice Teachers’ Health Literacy Levels and Teaching Practices: Protocol for a Design-Based Research Approach

**DOI:** 10.2196/69900

**Published:** 2025-11-12

**Authors:** Cassidy Kealy-Ashby, Louisa R Peralta, Wayne G Cotton

**Affiliations:** 1 University of Sydney Darlington, NB Australia

**Keywords:** health literacy, education, teacher education, preservice teachers, initial teacher education

## Abstract

**Background:**

Research examining the design and impact of health literacy (HL) programs and interventions on preservice teachers’ HL and health and physical education (HPE) teaching practices to enhance students’ HL is nonexistent. However, research indicates that many teachers and preservice teachers report low levels of HL, and recent research has highlighted preservice teachers’ challenges in teaching HPE to enhance their students’ HL capabilities. This is concerning, as HL is a focus in curricula and schooling both in Australia and internationally. Therefore, teachers are engaging in professional development aimed at enhancing their HL teaching practices, which raises questions about how further development of initial teacher education can enhance preservice teachers’ HL capabilities and teaching practices in schools.

**Objective:**

This protocol aims to provide details of a study to be conducted in the University of Sydney, involving final-year HPE preservice teachers, aimed at developing their HL capabilities and teaching practices.

**Methods:**

The proposed methodology underpinning the research program follows a 7-stage design-based research approach, with the 7 stages outlined in 4 sequential studies. These 4 studies include a systematic review, a qualitative focus group study, a pilot study, and a controlled trial study. This paper details the plan for conducting the 4 studies in a comprehensive and transparent manner, thus contributing to the methodological evidence base in this field.

**Results:**

The pilot study was conducted between July 2024 and December 2024, and data analysis was conducted between January 2025 and June 2025. The controlled trial will be conducted between July 2025 and December 2025. Final data analysis will occur between January 2026 and June 2026.

**Conclusions:**

This project will assist in determining what key stakeholders see as important to include in an initial teacher education program designed to enhance HPE preservice teachers’ HL capabilities and teaching practices. The design principles generated from this project will contribute to the inclusion and development of initial teacher education programs and practices and strengthen preservice teachers’ HL and their teaching of HPE, thereby enhancing HL teaching practices. This is significant considering that HL is included in the curriculum internationally, and the development of HL for school-age students has been identified as a World Health Organization goal.

**International Registered Report Identifier (IRRID):**

DERR1-10.2196/69900

## Introduction

### Background

Health literacy (HL) is a relatively new phenomenon in the field of education [1,2], despite being a concept used in the health and medical field for at least 3 decades. HL was first mentioned in 1974 [3] and was further conceptualized in the health and medical field, as it has been recognized as a cognitive skill leading to positive health outcomes, particularly a reduction in lifestyle diseases and an increased ability to navigate the health care system [4-6]. Over the last 3 decades, HL research has found that higher HL levels are associated with decreased government spending, less frequent hospital visits, and increased patient knowledge and understanding [7,8].

More recently, greater focus has been placed on electronic HL (eHL). eHL can be defined as “the ability to seek, find, understand, and appraise health information from electronic sources and apply the knowledge gained to addressing or solving a health problem” [9]. This rise in eHL research has been in response to young people spending a greater amount of time online and on social media [10], which was seen to be particularly relevant in the COVID-19 pandemic [11]. This, alongside growing digitalization, has seen an infodemic of health information and misinformation, making it difficult for people to navigate and understand health resources online [12].

The social determinants of health are the conditions in which people are born, grow, live, work, and age and include a range of factors that influence health [13]—many of which youth have limited control over (eg, food security). In recent years, much of the research in the field of HL has focused on investigating whether HL is an independent determinant of health [14-16]. Large-scale research studies that used the European HL Survey across 8 European countries [14,15] supported that HL is a “relevant, independent, direct determinant of self-assessed health” [17]. HL is now being recognized as a social determinant [18]; this highlights the importance of creating systems for youth that develop and enhance HL knowledge and capabilities.

In recent years, HL has been reconceptualized to indicate the complex relationships among the individual, school education, community, and the health services they seek to access. Therefore, HL is not considered to be learned through only 1 system, setting, structure, or relationship [16,18]. The understanding of HL has evolved to include HL of two interrelated components: (1) individual HL and (2) the HL environment [19]. Individual HL “is the skills, knowledge, motivation and capacity of a person to access, understand, appraise and apply information to make effective decisions about health and health care and take appropriate action” [19], while the HL environment “is the infrastructure, policies, processes, materials, people and relationships that make up the system and have an impact on the way that people access, understand, appraise and apply health-related information and services” [19]. Both dimensions are important to consider in current HL research, particularly for this study, as preservice teachers are likely to have developed and will continue to develop their own HL through a combination of personal and professional experiences, including formal university education.

HL plays a critical role in empowering an individual [20], which leads to several countries including HL in their national documents and goals. The World Health Organization published an HL report in 2013, making explicit recommendations to strengthen HL levels of school-age children [21]. In response to this, HL was then included in the school curricula both in Australia [22] and internationally as well as in the education policies [23-25]. The current Australian health and physical education (HPE) curriculum [26] and the New South Wales (NSW) Personal Development, Health and Physical Education syllabus [27] (which will be referred to as HPE throughout this paper) have both adopted HL through HL being named as one of the 5 underpinning propositions, using 3-level hierarchal model proposed by Nutbeam [28]. The definition formulated by Nutbeam [28] is commonly used across international curricula and identifies HL in a hierarchical model, with 3 dimensions (functional, interactive, and critical HL), with each level being more advanced in terms of health decisions and impact on health outcomes. Functional HL is the first of the 3 dimensions and is recognized as the ability to use basic reading and writing skills to understand health information as well as apply instructions given by a physician, such as submitting a prescription or taking medication. Slightly more advanced is the interactive dimension, where individuals can apply health information in different contexts. The third dimension is critical, defined as “the cognitive skill and development outcomes which are oriented towards supporting effective social and political action, as well as individual action” [28]. When applied to classroom settings, the focus is on moving students toward being able to engage in community action, advocacy, critical thinking, analysis of policies, and engagement with leaders (ie, critical HL).

The ability of schools, school principals, other school-based leaders, and teachers to advance HL through curriculum, teaching, and learning across the whole school environment is central to the implementation of HL in schools and the promotion of HL for children and adolescents [29-32]. Therefore, the teacher plays a key role in developing students’ HL levels [33-35]. Despite the importance of the teacher’s role, research shows that Australian teachers (1) have insufficient understanding of how to best develop HL [30] and (2) only have access to limited professional development programs that focus on the promotion of HL [29,30,32]. These findings from Australian settings are in line with the findings from international studies. A cross-sectional study of 520 schoolteachers in an education zone in Colombo, Sri Lanka, found that 33% of the teachers fell into the “limited” HL category, as determined by the European Union HL survey [4]. The study by Denuwara and Gunawardena [4] also found that teachers who were aged 45 years or younger were associated with lower HL levels compared to their older colleagues. Furthermore, another study [36] found that 74% of the Turkish primary teachers have low or limited levels of HL. In response to this need to increase teachers’ HL levels, many schools have engaged in teachers’ professional development [29,30,32,35] and adopted structural changes such as a whole-school approach to developing HL [35]. Although the evidence suggests that these approaches are effective, they require significant systemic changes and are costly in terms of time and finances [37]. Therefore, this poses questions about the roles of universities in developing preservice teachers’ HL and their ability to teach HL through initial teacher education programs.

One study conducted in Indonesia [38] found that of the 704 biology preservice teachers, 53% were identified as having low levels of HL. The study, which focused on HL levels in relation to COVID-19 information, concluded with revealing important findings that suggested that preservice teachers had limited capabilities when it comes to analyzing and evaluating health-related information [38]. The 2 main limitations of the study were its focus on COVID-19 HL and not exploring preservice teachers’ teaching of HL. Two recent studies conducted in Australia used a set of data to compare the HL levels of final-year primary generalist and secondary specialist HPE preservice teachers with one another and with the general population [39,40]. The studies examined HL levels using the reliable and valid HL Questionnaire (HLQ) [41] and found that HL levels were lower among primary generalist preservice teachers than among HPE specialist preservice teachers across all 9 sections of the HLQ. Interestingly, when compared to the general Australian population, the results showed that primary generalist preservice teachers had lower HL levels across domains 8 to 9 of the HLQ, indicating lower HL representative of functional and interactive HL [39]. This finding is significant, considering both primary and HPE teachers have a responsibility to teach HL within HPE as per the Australian curriculum [26] and NSW syllabus [27]. The first study then discussed possible reasons for these differences, using interview data and a document analysis of the university’s scope and sequences for both programs, highlighting that the HPE cohort spent considerably more time in health-related units than the primary cohort. From this, it can be gathered that the more time spent in initial teacher education units as well as more explicit teaching of HL, including its key concepts, could possibly be connected to higher HL levels and perceptions of teaching HL. Data obtained from interviews with the primary preservice teachers’ cohort revealed that they wanted more time spent on HL development and more explicit mention of HL in their only initial teacher education unit. Interviews also highlighted concerns in teaching HL in an increasingly digital world, acknowledging the importance of eHL development. Interestingly, although the HPE cohort consisted of individuals who were specialists in teaching HPE, participants still expressed concern about teaching HPE to enhance students’ critical HL, indicating the need for redesigning and restructuring initial teacher education units in initial teacher education programs to consider these suggestions [39,40].

Despite recent research highlighting a need for preservice teachers’ HL development, there has been no known HL intervention for promoting preservice teachers’ HL and HL teaching. Much of the research on HL intervention programs exists in the clinical setting [42,43]. Although these studies generally focus on enhancing a practitioner’s HL, the principles may be applied and adapted to suit broader university settings. The commonality between studies indicates the significance of realistic and explicit scenario-based learning [42,44,45], which can be achieved through internet access to develop digital HL skills [45,46]. Kripalani and Weiss [43] highlighted the significance of modeling within programs, including modeling clear communication and teach-back techniques, which may be useful to replicate in a classroom setting. The research conducted by Mogford et al [45] suggested interactive pedagogy and critical consciousness strategies to create an HL intervention for university programs but not specific to initial teacher education. The research recommended structuring a university’s initial teacher education unit to best develop critical HL by dividing the program into 4 parts: *knowledge, compass, skills,* and *action.* However, the researchers have yet to conduct pretesting and posttesting to determine whether these parts have an impact on university students’ HL. Nonetheless, these suggestions could be used to inform the co-design and refinement of an HL intervention for preservice teachers.

### Objectives

To the best of our knowledge, no study has reported on the design, implementation, and impact of an intervention program on developing preservice teachers’ HL levels and enhancing HL teaching practices. This study aims to fill this research gap. The aim of this study is to co-design an HL intervention program in a unit of study in 1 initial teacher education program at the University of Sydney. Using a design-based research (DBR) approach, embedding a pilot study and controlled trial (CT), a series of design principles will be created that will be evidence informed and can be used to underpin the design of future initial teacher education units and programs.

This project will investigate how HL curriculum goals can be implemented in initial teacher education programs to enhance HPE preservice teachers’ HL and eHL teaching practices. To address this overarching research question, this study will examine the extent to which a unit of study in an initial teacher education program may impact preservice teachers’ HL and eHL levels and the extent to which it influences preservice teachers’ teaching of HPE to enhance HL and eHL teaching practices.

### Theoretical Framework

The socioecological model [47] underpins this study as it examines the interrelationship between individuals and groups and assesses the way that these relationships exist within a broader social network or ecology [48]. The framework adopts a social constructivist approach [49,50], which is the idea that knowledge is formed in collaboration with others [49]. Wharf Higgins et al [50] argued that although health behaviors are perceived as outcomes of cognitive decisions, this view is limited because intrapersonal, interpersonal, and community levels all influence an individual’s health knowledge, behaviors, and capabilities. In the same way, HL is socially constructed, with influences of the micro, meso, and macro contexts, determining a person’s HL level. Using the socioecological model proposed by Bronfenbrenner [48], this study will consider the interaction between the micro, meso, and macro contexts, which is important because it recognizes that universities play a significant role in developing an individual’s HL and the teaching of HL through HPE in schools to enhance students’ HL. This framework will be used in this study by ensuring that quantitative data on HL and eHL levels are triangulated with qualitative data to determine how the experiences of engaging with the health education unit may have influenced the development of preservice teachers’ HL and eHL capabilities and teaching practices.

## Methods

### Research Design and Procedure

#### Overview

**Figure 1:**
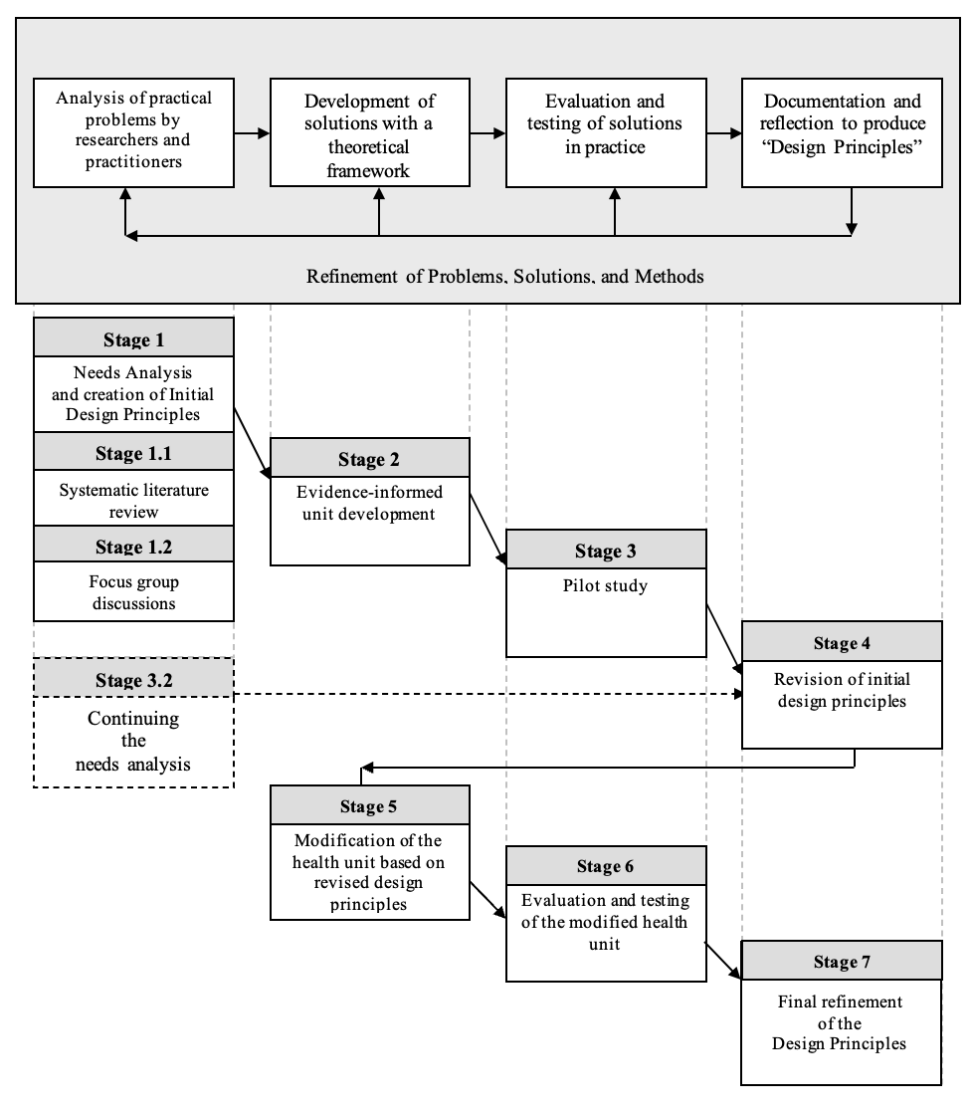
A diagrammatic overview showing how the proposed research aligns with the design-based research model proposed by Reeves [51] (adapted from Sultoni et al [52], which is published under Creative Commons Attribution 4.0 International License [57]).

To investigate the developmental nature of the research questions, a robust methodological framework is required. Reeves’ [51] original design-based research model will be used to underpin the research methodology [52] for this study (Figure 1). The justification for selecting a DBR model is that, at its core, DBR assumes real-world practice is contextual; hence, for effectiveness, an applied practice must be flexible to the needs of the group [52,53]. The study will focus on the premises of the model, which is needed to move beyond the limited measures of learning to address the nature of learning in the real world [54]. This is important considering university initial teacher education programs exist predominantly in the mesocycle of the socioecological model [49]. The DBR model, in recent years, has been applied to complex education intervention studies [55,56], divided into 4 key stages. For this study, a 7-stage process has been developed to reflect the changing nature of the design, which underpins this study.

#### Stage 1: Needs Analysis and Creation of Initial Design Principles

##### Overview

The aim of stage 1 is to conduct a detailed needs analysis of the problem to inform the creation of an initial series of design principles. These initial design principles will be used in stage 2 to inform the co-design of an existing initial teacher education unit in a Bachelor of Education (HPE) program. The needs analysis will involve two steps: (1) a systematic literature review will be conducted to explore current research on an analysis of features and characteristics of university HL and eHL interventions and (2) a series of semistructured interviews will be conducted to further develop the findings of the systematic literature review and situate them in an Australian and NSW initial teacher education contexts. These steps are described in detail subsequently.

##### Stage 1.1: Systematic Literature Review

The aim of the systematic literature review is to determine the need for an HL and eHL intervention and the features and characteristics to be included within the initial teacher education unit. The research will examine, appraise, and provide recommendations from the available literature to support what could form an impactful initial teacher education unit (ie, one that has the potential to enhance HL among preservice teachers). This systematic review will follow the PRISMA (Preferred Reporting Items for Systematic Reviews and Meta-Analyses) guidelines [58], and the protocol of the study will be registered with PROSPERO. The review will be informed by the Cochrane Handbook for Effective Systematic Literature Reviews [42]. This will include identifying studies relevant to this research, collating the data, considering the statistical analysis, and interpreting the results. The population, intervention, comparison, and outcome method will be used for determining the search terms for the systematic literature review, as this has been identified as an effective way to align with research questions for health-based interventions [59]. The population will consist of university students or higher or further education students enrolled in teacher education courses or health courses. Intervention will include programs aimed at developing HL and eHL levels using key strategies. Comparison will be between a study that includes a CT and a study involving usual control methods. The primary outcomes will be HL and eHL as well as HL teaching practices embedded within HPE.

More specifically, database fields will be searched using the terms provided in Textbox 1.

**Database field search strategy.**

- Population: Universit* OR college OR TAFE

AND

- Intervention: health literac*

AND

- Comparison: “control trial”* OR “control group”* OR RCT* OR quasi* OR “CT” OR experimental* OR pre* OR post* OR evaluat*

AND

- Outcomes: “Health literac”* OR teach* OR school* OR students* OR peers* OR universit* OR “health education” OR HLQ OR hosp* OR patient*

The databases that will be searched include MEDLINE, Embase, The Cochrane Library, PubMed, PsycINFO, and EBSCOhost. These databases have been selected based on existing systematic literature reviews and literature reviews in the field of HL and health education [14,46,60]. Reference lists from included articles will also be searched to identify and examine other relevant studies for the systematic literature review. Data from 1974 until mid-2023 will be reviewed, as 1974 was the year the term HL was first published in education literature [3]. Results will be limited to peer-reviewed articles written in English. Stage 1.1 will inform the first publication.

##### Stage 1.2: Semistructured Interviews With Key Stakeholders

The aim of the semistructured interviews is to further develop an analysis of the practical problems by including practitioners to inform and validate the initial design principles revealed in stage 1.1. The interviews will be conducted via Zoom (Zoom Communications, Inc) [61]. The participants for stage 1.2 will include the unit coordinator and lecturers or tutors of the final-year initial teacher education unit at the university, 9 other lecturers or tutors of HPE preservice teachers at other Australian universities, and 2 early-career HPE teachers (n=11). The semistructured interviews will situate the findings from the systematic literature review in an NSW educational context. The discussions will also help to develop a better understanding of what is needed for effective HL and eHL development, how this knowledge will transfer into schools, and what preservice teachers need to feel most confident in teaching HPE to enhance students’ HL and eHL from the perspective of key stakeholders.

The face-to-face interview recordings will be transcribed by the lead researcher, and the online interviews will be transcribed from Zoom interview recordings. To analyze the interview data, we will follow an inductive approach in the qualitative research to ensure that it is exploratory rather than guided by particular themes [62]. The reflexive thematic data analysis model by Braun and Clarke [63] will be adopted. A reflexive approach to thematic analysis has been considered as it acknowledges the researchers’ subjectivity as a resource that can analyze the data [63]. This is because the researcher, as a student, has completed the unit that is going to be co-designed and refined. Therefore, coding of these interviews will be conducted in parallel with another researcher not involved in the unit, to reduce bias, as well as following the reflexive approach of the researcher. The process consists of familiarizing oneself by writing notes on the transcripts. Codes will then be used to summarize portions of the data. Once coded, themes will be compared by examining the similarities between the codes from the previous step to find thematic concerns. Then, the boundaries of the themes will be considered to determine whether there are enough data to support them. Once themes are defined and named, they will be analyzed deductively using the socioecological framework. The findings from this stage will further establish or add to the initial design principles established through the systematic literature review. An initial outline of the semistructured interview questions (for a head of the HPE department in an NSW secondary school) can be found in Multimedia Appendix 1. This stage will inform the second publication. Ultimately, the results of the systematic literature review and the semistructured interviews will form the initial design principles to be used to co-design the existing initial teacher education unit.

#### Stage 2: Evidence-Informed Initial Teacher Education Unit Development

The aim of stage 2 is to develop solutions, based on stage 1, within the socioecological framework. To do this, the initial design principles and data from stage 1 will be used in conjunction with the initial teacher education unit of study coordinator and lecturer to co-design the unit of study.

The initial teacher education unit is currently scheduled for the final year of an HPE course at the university and focuses on health promotion in community health issues. It is a 6-credit point unit (equates to one-eighth of a full-time student load) that runs for 8 weeks and is compulsory for all final-year HPE preservice teachers. The unit runs parallel with the final-year professional experience unit, which requires preservice teachers to complete their final school placement and consequently complete their teaching performance assessment (TPA), which is completed at this university as the Assessment for Graduate Teaching (AfGT) [64].

Intervention programs in initial teacher education co-designed with stakeholders have proven to be more efficient than informing an intervention based solely on the dissemination of information [65]. In this study, a co-design approach has been selected, using a participatory approach that relies on the knowledge of stakeholders, which is common in the health education field [66]. Co-design that includes the ideas of stakeholders is more likely to be accepted, adopted, and sustained [67]. Both the systematic literature review and the semistructured interview data, through the initial design principles, will be used to co-design the final-year initial teacher education unit with the unit coordinator. This process will be based on the same rationale, outcomes, structure, and assessment type. The process will include content development, which aims to produce modules for the initial teacher education unit, tutorial and assessment activities, provocation questions, scenario-based learning, and tutorial tasks.

#### Stage 3: Testing of the Developed Initial Teacher Education Unit

##### Overview

The aim of stage 3 is to evaluate and test the solutions in practice, that is, the impact of the co-designed unit of study on HL levels and the teaching of HL. Specifically, this stage will determine the feasibility, acceptability, and potential efficacy of a CT in a real-world setting to assess the 3 primary outcomes (preservice teachers’ HL levels, preservice teachers’ teaching practices in HPE and HL in coursework, and HL teaching practices) as outlined subsequently. Testing the feasibility of an intervention program focuses on increasing the validity of the intervention by deciding whether key factors, including timelines, recruitment, and delivery, are feasible before conducting a CT [68]. To test acceptability, qualitative interview data will be collected to determine the extent to which the intervention program is acceptable to the target population and suitable for their needs [69]. To ensure bias is removed, the questions for these interviews have been created by the lead researcher, who is also the lecturer and tutor, alongside researchers who are not engaged with the cohort and are therefore possible participants.

The testing of the co-designed unit of study will therefore be conducted. To do this, the participants of the pilot study will include the final-year HPE cohort at the university in their initial teacher education unit [70]. There will be approximately 30 preservice teachers in the class. In addition to the primary outcomes mentioned subsequently, the feasibility and acceptability of the co-designed unit of study will be determined by interviewing the lecturer through a semistructured interview (n=1) and obtaining consent from preservice teachers (n=5) in a focus group discussion to identify whether the unit achieved the unit outcomes, the feasibility of the content and learning experiences, and how the unit was conducted in a realistic setting. An overview of the semistructured interview questions is provided in Multimedia Appendix 2. An overview of the focus group discussion questions is provided in Multimedia Appendix 3.

To triangulate the data, patterns of convergence or divergence between quantitative results (eg, questionnaire data) and qualitative findings (eg, interviews) will be identified. This process will assist in enhancing the coherence of the study and assist in coming to practical conclusions for the initial design principles. The primary outcomes for this stage are mentioned subsequently.

##### Stage 3.1: Preservice Teachers’ HL and eHL Levels

HL will be measured objectively using the HLQ [41]. The HLQ will be administered in week 1 and again at the conclusion of the unit in week 8. Data will be analyzed using protocols developed in previous studies [40,41,70]. This will include using SPSS (version 28; IBM Corp) for analysis of HLQ scores, applying robust ANOVA with the Welch method. The selected HLQ assesses HL as a multidimensional concept categorized in 9 sections rather than producing a single score, hence offering a broad and valid understanding of a person’s HL capabilities. The 9 sections have been deemed valid for young adults [41] and reliable, with the composite reliability ranging from 0.8 to 0.9 [41,70]. To measure eHL levels, the University of Copenhagen’s eHealth Literacy Questionnaire [71] will be used. This multidimensional tool assesses eHL across 7 scales and does not generate a single score but rather provides a broader understanding of eHL levels across different areas and scales. For this, the same analysis process as the HLQ will be used, as mentioned earlier. This will address the research question “To what extent can a unit of study impact preservice teachers’ HL and eHL levels?” These findings will assist in answering the key question of the study and achieving the main aim of the co-designed unit, which is to enhance preservice teachers’ HL and eHL levels, making this the most prioritized outcome of the study.

##### Stage 3.2: Preservice Teachers’ Teaching Practices in HPE and HL in Coursework

Teaching practices will be assessed through work samples created in the initial teacher education unit. Data will be collected at the end of each tutorial and through the assessment tasks. These will be annotated by both the lead researcher and an additional researcher not involved with the cohort to identify how HL concepts have been applied in different contexts, which is important because research indicates that HL must be adjusted to suit different settings and educational goals [33]. In line with the Australian HPE curriculum and the HL proposition in the NSW Personal Development, Health and Physical Education syllabus [26-28], the 3-level hierarchical model proposed by Nutbeam [28] (functional, interactive, and critical) will be used to analyze the application of HL to health-related coursework tasks. The work will be deductively analyzed to determine where it is situated in relation to the hierarchical model [28]. This process is commonly used in other research projects in the field that examine evidence of HL content and teaching [32,72]. By analyzing the data deductively, this study aims to assess how preservice teachers plan for HPE teaching and learning that will enhance their students’ HL levels. This outcome will address both subsidiary research questions.

##### Stage 3.3: HL Teaching Practices

This outcome will be measured using data from preservice teachers’ final-year professional experience placement, a supervised working placement, administered in a parallel unit of study. This is an important outcome as it will assist in determining if the preservice teachers can transfer their HL and eHL learning experiences into their health education classes and whether students can, as a result, develop their own HL and eHL. The sample size will be the number of consenting students who choose health lessons (n=20). The data collected will be the participating students’ completed AfGT. The AfGT has been approved by the Australian Institute for Teaching and School Leadership [72] expert advisory group and deemed valid as a TPA, having been developed to assess practice and competency, aligning with the national program standard at the graduate level. Data will be analyzed using the same process in stage 3.2, with deductive analysis to determine if and how the teaching and learning strategies have applied HL according to Nutbeam’s hierarchy [28].

Follow-up interviews with preservice teachers (n=5) will also be conducted to reflect and triangulate the evidence from the TPA. The AfGT will be used as a discussion point for the interviews, and participants will be encouraged to bring additional artifacts (eg, additional lesson plans, lesson resources, or work samples) from their internship for further evidence. Sample semistructured interview questions are provided in Multimedia Appendix 4. Interview questions were selected to assist in measuring quality and improvements in teaching, as professional reasoning is closely linked to teaching professionalism [73]. The same interview analysis process will be conducted, as explained in stage 1.2. Both primary outcomes 2 and 3 will be used to answer the following research question: “To what extent can a unit of study influence preservice teacher’s teaching of HPE to enhance HL?” Stage 3 will inform the third publication.

#### Stage 4: Revision of Initial Design Principles

The aim of stage 4 is to focus on documentation and reflection to produce a revised set of design principles. To revise the design principles, the proposed strategy is to collate the data from stage 3 and refine accordingly using the data from stage 3.2. These revised principles, with evidence-based modifications, are necessary to modify the initial teacher education unit in stage 5.

#### Stage 5: Modification of the Initial Teacher Education Unit Based on Revised Design Principles

The aim of stage 5 is to continue with the development of solutions. This will be the modification of the initial teacher education unit of study based on the revised design principles. To do this, the initial teacher education unit of study will be further co-designed with the unit coordinator. This will follow the same rationale, outcomes, structure, and assessment type. The process will include making changes based on the pilot study and revision of design principles.

#### Stage 6: Evaluation and Testing of the Modified Initial Teacher Education Unit

The aim of stage 6 is to re-evaluate and test the revised solution in practice. The location of this stage will be at The University of Sydney and/or The Australian Catholic University and The University of Tasmania. The modified health unit will be tested through a CT, including the final-year HPE cohort (n=30) from the university and another Australian university (n=30). Although not often used in education, a CT has the advantage of providing a more precise estimate of effect when comparing 2 educational groups, one of which receives an intervention program [74]. The lecturer (n=1) in the intervention group will conduct the modified unit while the other university final-year cohort will deliver their usual unit (with no changes implemented). To enhance validity, baseline information will be collected from both cohorts using a demographic data questionnaire and a pretest score for their HLQ. To reduce the risk of contamination, both universities will conduct their courses independently without communicating about content or sharing resources. Data will be gathered using the valid and reliable instruments administered in stage 3 to complete preintervention and postintervention research on both groups. A series of 2-tailed *t* tests will also be conducted for the HLQ. Coding of the artifacts will be compared as well. Data will be analyzed following the same procedure used in stage 3. A power analysis will be conducted to determine whether the sample size is sufficient to detect statistically significant changes. If it is detected to be underpowered, this will be identified in the publication as a limitation of stage 6. Stage 6 will inform the fourth publication.

#### Stage 7: Final Refinement of the Design Section Principles

The aim of the final stage (stage 7) of the study is to use the data collected between stages 1 to 6 to create a concluding series of design principles to assist future initial teacher education program leaders, unit of study designers, initial teacher education lecturers, and tutors in initial teacher education who coordinate and teach within HPE.

### Ethical Considerations

Ethics approval and consent to participate were sought and accepted from the University of Sydney human research integrity and ethics committee for this study (2024/081). Participants who consented to the study will be made aware that their data will remain anonymous and any interview data will be de-identified. However, the consent form did indicate that if participants wished to withdraw from the HLQ and eHLQ, their data would not be removed as it was anonymous and therefore the researchers cannot distinguish their data from other participants.

## Results

Stages 1.1 and 1.2 were conducted between January 2024 and May 2024, and the results have been submitted for publication. Stage 2 was conducted between June 2024 and July 2024, and stage 3, being the pilot study, was conducted between July 2024 and December 2024. Data analysis was performed between January 2025 and June 2025. The CT will be conducted between July 2025 and December 2025. Final data analysis will be conducted between January 2026 and June 2026.

## Discussion

### Anticipated Findings

The anticipated findings are an increase in HL and eHL levels of the preservice teachers who participated in the refined health education unit. The interview data are expected to contribute to the understanding of the preservice teachers’ perceptions of the unit and their confidence in planning and teaching HL learning experiences. By collecting these pretest and posttest data as well as the data from the interviews with key stakeholders, this study will assist in the development of the initial design principles. The purpose of this study is to evaluate the impact of a final-year HPE HL and eHL intervention and the extent to which it is transferred into learning and curriculum goals in the classroom. The intervention uses a DBR approach to determine the current HL and eHL intervention programs used in the university setting as well as recommendations for the co-design process from the perspective of key stakeholders. Determining these design principles will help support the co-design of the initial teacher education unit aimed at enhancing HL levels and HL teaching practices. This paper describes the systematic creation of a series of design principles using literature, semistructured interviews with key stakeholders, and the final principles modified through the pilot testing process. Findings from this study are expected to contribute to initial teacher education programs internationally and inform the design process of the initial teacher education units.

### Comparison to Prior Work

Studies have identified preservice teachers’ low levels of HL and eHL and recognized a need for development of these capabilities [39,40]. However, there is no published intervention that focuses on the development of preservice teachers’ HL and eHL levels. Therefore, this research will extend previous research by applying a DBR approach to co-design a health education unit and implement and evaluate the potential impact of this unit on HL and eHL levels and teaching practices.

### Strengths and Limitations

The key strength of this study is its novel contribution to the field. Other strengths include the DBR within a mixed methods approach, the use of validated instruments, and artifact (AfGT) coding, along with qualitative interviews, to triangulate the data. However, there are also some limitations to be acknowledged, including the small sample size (n=30), which may impact the statistical power of this study. In addition, because this study was initially conducted in a single Australian university, the CT will be necessary to provide stronger evidence of impact. As the CT will include an additional university, the groups will not share data or resources; however, some contextual and demographic differences between the 2 universities may remain.

### Future Directions

Findings from this study, particularly the initial design principles informed by literature and key stakeholder input, will inform the development of other health education programs aimed at developing HL and eHL levels and teaching practices. Although this study will determine whether HL and eHL practices can be transferred into teaching, future research may broaden the context of the health education units’ delivery, refining some elements to test the suitability of the design principles in other contexts, such as in the medical field or allied health.

### Dissemination Plan

Results from this study will be disseminated through peer-reviewed journal publications and conference presentations. The final design principles will be shared with key stakeholders involved in the co-design process, allowing them to potentially include these design principles in their health education programs, which would enhance the broader impact of the study.

## Appendix

Multimedia Appendix 1 Example of semistructured interview questions for stage 1.1—semistructured interviews with key stakeholders.

Multimedia Appendix 2 Example of semistructured interview questions for stage 3—lecturers.

Multimedia Appendix 3 Example of focus group interview questions for stage 3—preservice teachers.

Multimedia Appendix 4 Example of semistructured interview questions for stage 3—preservice teachers.

## Abbreviations

AfGT: Assessment for Graduate Teaching
CT: controlled trial
DBR: design-based research
eHL: electronic health literacy
HL: health literacy
HLQ: Health Literacy Questionnaire
HPE: health and physical education
NSW: New South Wales
PRISMA: Preferred Reporting Items for Systematic Reviews and Meta-Analyses
TPA: teaching performance assessment
