# Supplementary material for: Health and Physical Education Preservice Teachers’ Health Literacy Levels and Teaching Practices: Protocol for a Design-Based Research Approach
Source: JMIR Res Protoc. 2025 Nov 12;14:e69900. doi: 10.2196/69900 (PMC12658394; doi:10.2196/69900)
Supplement: Multimedia Appendix 1 [file resprot_v14i1e69900_app1.docx]

This interview is part of a PhD research project that focuses on developing health literacy levels and teaching practices amongst final year PDHPE Preservice teachers. Talking about the initial design principles today and getting an understanding of your views on these design principles will help to co-design a unit to develop Preservice teachers’ health literacy and teaching practices. You have been invited to participate in this research study as you are a key stakeholder in schools.

This is the start of this interview. Please respond to the questions as honestly as you can, as there are no right or wrong answers and no trick questions. Your responses will be held in confidence and only used for research purposes.

- What role does a PDHPE teacher play in developing their student’s health literacy levels?
- What do you believe are the biggest barriers to effective health literacy teaching in high schools?
- How could these barriers be overcome?
- What do you think the greatest challenges high schools are now facing in terms of student’s health decisions?
- What makes for effective health literacy teaching?
- What skills do you think graduate teachers should have to be able to teach health literacy effectively?
- What do schools currently need to enhance the health literacy of their students?
- Is there anything universities could do to better prepare PDHPE teachers to develop their student’s health literacy capabilities? If so, what are they?

We have conducted a systematic review of the literature, and these are the initial design principles.

- Based on your experience in your specific field, do you agree with these initial design principles? Why/ why not?
- What are the potential positive elements of the initial design principles?
- What are some of the potential negative elements of the initial design principles?
- Is there anything that you believe is missing in the initial design principles and should be included? If so, what is it and why?
