# Supplementary material for: Health and Physical Education Preservice Teachers’ Health Literacy Levels and Teaching Practices: Protocol for a Design-Based Research Approach
Source: JMIR Res Protoc. 2025 Nov 12;14:e69900. doi: 10.2196/69900 (PMC12658394; doi:10.2196/69900)
Supplement: Multimedia Appendix 2 [file resprot_v14i1e69900_app2.docx]

## Example of semi structured interview questions for Stage 3: Lecturer

This interview is part of a PhD research project that focuses on developing health literacy levels and teaching, amongst final year PDHPE preservice teachers. You have been invited to participate in this research study as you taught the unit.

This is the start of this interview. In this interview, we are interested in your perceptions of the unit, its feasibility and any suggestions you have. Please respond to the questions as honestly as you can, as there are no right or wrong answers and no trick questions. Your responses will be held in confidence and only used for research purposes.

- Do you believe that the unit of study was effective in developing preservice teachers’ health literacy levels? Why/ why not?
- Was there the correct amount of content in the unit for it to be realistically carried out across 8 weeks? Why/ why not?
- To what extent did the activities and assessments meet the outcomes?
- How effective were the assessments in developing health literacy skills?
- What teaching strategies worked best within the unit to develop health literacy and what did not work as well?
- In your opinion, how did the students engage with the unit and its content?
- Is there anything noteworthy that should be considered for the refinement of the unit for the future?
