# Supplementary material for: Health and Physical Education Preservice Teachers’ Health Literacy Levels and Teaching Practices: Protocol for a Design-Based Research Approach
Source: JMIR Res Protoc. 2025 Nov 12;14:e69900. doi: 10.2196/69900 (PMC12658394; doi:10.2196/69900)
Supplement: Multimedia Appendix 3 [file resprot_v14i1e69900_app3.docx]

## Example of focus group interview questions for Stage 3: Preservice teachers

This interview is part of a PhD research project that focuses on developing health literacy levels and teaching practices, amongst final year PDHPE preservice teachers. You have all been invited to participate in this research study as you have participated in the unit.

This is the start of this discussion. In this discussion, we are interested in your perceptions of the unit, its feasibility and any suggestions you have. Please respond to the questions as honestly as you can, as there are no right or wrong answers and no trick questions. Your responses will be held in confidence and only used for research purposes.

- Do you believe that the unit impacted your health literacy levels? How?
- I will give you a moment to reflect on the activities that you completed throughout the tutorials. Can you recall any specific tutorial activities that helped to develop your health literacy levels or your health literacy teaching practices? If so, what specifically did you think was effective about them?
- Have a think about your assessments in the unit. Did any elements of the assessment/s challenge you to improve your health literacy?
- Having a look at the unit outcomes, do you believe that these were met? Why/ why not?
- Do you believe that there was the correct amount of content for the 8-week unit? Why/ why not?
- After completing the unit, how confident do you feel in your teaching practices to develop your student’s health literacy skills?
- Are there any suggestions that we should consider for the refinement of the unit? If so, what are they and why?
