# Supplementary material for: Health and Physical Education Preservice Teachers’ Health Literacy Levels and Teaching Practices: Protocol for a Design-Based Research Approach
Source: JMIR Res Protoc. 2025 Nov 12;14:e69900. doi: 10.2196/69900 (PMC12658394; doi:10.2196/69900)
Supplement: Multimedia Appendix 4 [file resprot_v14i1e69900_app4.docx]

## Example of semi structured interview questions for Stage 3: preservice teachers

This interview is part of a PhD research project that focuses on developing health literacy levels and teaching practices, amongst final year PDHPE Preservice teachers. You have been invited to participate in this research study as you have participated in the unit. We are interested in understanding your thought processes behind planning and implementing your teaching practices to develop students’ health literacy levels on your internship. In this interview, we will be referring to your AfGT as well as any other artefacts you may have chosen to bring with you today. Please address these where possible in your responses.

This is the start of the interview. Please respond to the questions as honestly as you can, as there are no right or wrong answers and no trick questions. Your responses will be held in confidence and only used for research purposes.

- When planning your lessons, what were your thought processes in trying to enhance your student’s health literacy skills?
- Throughout your internship, what teaching strategies did you use to try to enhance your students’ health literacy skills?
- Looking at your assessment data (including formative, summative and diagnostic), did your assessments assess health literacy levels of your students? If so, what were the findings?
- Did you use any resources, learning opportunities or activities from your final year health education unit in your planning and implementation of teaching practices?
- What did you find proved the greatest challenge in your health literacy teaching practices?
- Is there anything that could have been included to improve the final year health education unit to better prepare you to teach health literacy? If so, what?
